# Spatial Genome Organization: From Development to Disease

**DOI:** 10.3389/fcell.2019.00018

**Published:** 2019-03-21

**Authors:** Aishwarya Sivakumar, Jose I. de las Heras, Eric C. Schirmer

**Affiliations:** Wellcome Centre for Cell Biology, University of Edinburgh, Edinburgh, United Kingdom

**Keywords:** genome organization, LAD, TAD, CTCF, cohesin, development

## Abstract

Every living organism, from bacteria to humans, contains DNA encoding anything from a few hundred genes in intracellular parasites such as *Mycoplasma*, up to several tens of thousands in many higher organisms. The first observations indicating that the nucleus had some kind of organization were made over a hundred years ago. Understanding of its significance is both limited and aided by the development of techniques, in particular electron microscopy, fluorescence *in situ* hybridization, DamID and most recently HiC. As our knowledge about genome organization grows, it becomes apparent that the mechanisms are conserved in evolution, even if the individual players may vary. These mechanisms involve DNA binding proteins such as histones, and a number of architectural proteins, some of which are very much conserved, with some others having diversified and multiplied, acquiring specific regulatory functions. In this review we will look at the principles of genome organization in a hierarchical manner, from DNA packaging to higher order genome associations such as TADs, and the significance of radial positioning of genomic loci. We will then elaborate on the dynamics of genome organization during development, and how genome architecture plays an important role in cell fate determination. Finally, we will discuss how misregulation can be a factor in human disease.

## Introduction

Over 60 years have passed since Watson and Crick published their famous model for the double helix structure of DNA. According to their model, the length of 1 bp DNA is 3.4 Å (Watson and Crick, 1953). If DNA was linear, a mere 6 kb stretch would cover the entire 2 μm length of a prokaryotic cell and a 20 kb stretch, the diametric length of an average eukaryotic nucleus. And yet, an astounding 4 million bp genome is packaged within a humble *Escherichia coli* bacterium and three billion bp in a human nucleus (Blattner et al., 1997; Venter et al., 2001). How this genome is packaged without compromising its accessibility when required is of interest to the entire field of molecular genetics, more so because congenital and developmental disorders are now being linked to disruption of spatial genome organization.

Carl Rabl’s prediction of a preserved centromere-telomere orientation throughout the cell cycle and Boveri’s argument in favor of discrete “chromosome territories” based on his observations of blastomere nuclei of *Ascaris megacephala*, fueled the earliest ideas of functional compartmentalization of the nucleus (Rabl, 1885; Boveri, 1909). Both these observations were possible due to a combination of light microscopy advances that managed to achieve 1 μm resolution in the mid-1800s and the use of unique model organisms that enabled better distinction of chromosome regions. Models of higher-order 3D spatial genome organization became disfavored in the 1960s and 1970s, when electron microscope studies identified 10–30 nm chromatin fibers, shifting focus to local chromatin folding (Comings, 1968; Wischnitzer, 1973). However, subsequent development of fluorescence *in situ* hybridization using whole chromosome painting probes (Lichter et al., 1988) made studying 3D genome organization possible, presenting conclusive evidence for non-random chromosome positions and existence of higher-order organization (for detailed historical review please see Cremer and Cremer, 2010).

**FIGURE 1:**
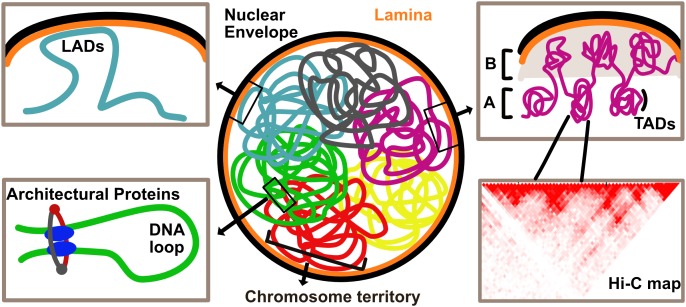
Overview of genome organization in the eukaryotic nucleus. Chromosomes are organized in discrete territories within the nucleus. Specific genomic loci called Lamina Associated Domains (LADs), detected by DamID, interact with the nuclear envelope and are typically repressed upon direct tethering. Topologically Associated Domains (TADs), defined by HiC, are units of the genome that frequently associate with each other. Higher order associations of TADs form A and B compartments which are typically enriched in transcriptionally active and inactive chromatin, respectively. Local chromatin loops are stabilized by architectural proteins such as CTCF and cohesin complex.

Drawing inferences from various techniques, we take a look at the hierarchy of genome organization, starting with the lowest units building up toward higher-order structures (Figure 1).

## Hierarchical Principles of Genome Organization

The most local level of chromatin folding and compaction is the wrapping of 146 bp around a nucleosome. These in turn fold to form the 10 and 30 nm fibers observed by EM.

### TADs, Loops and Compartments

Chromosome conformation capture, particularly its high throughput variant Hi-C, measures pair-wise DNA-DNA contact frequencies across the entire genome (Dekker et al., 2002; Lieberman-Aiden et al., 2009). This technique illuminated several *cis* and *trans* interactions, expanding our understanding of how DNA is folded in 3D and what biological meaning this folding confers. The first level of higher-order chromosome organization identified with this approach was the presence of megabase-sized blocks, called Topologically Associated Domains (TADs) (Figure 1). Interactions within TADs occur more frequently than those between TADs (Dixon et al., 2012). A subset of TADs contains “loop domains” which are special in that they can directly interact at their boundaries, thus forming a loop. In human cells, these loops are stabilized by CTCF, an architectural protein binding to the CCCTC motif, which is also found at TAD boundaries (Rao et al., 2014; Figure 1).

Genome organization and packaging into higher-order structures poses challenges with respect to protein accessibility to tightly packed regions. Local decondensation and chromatin looping is therefore required to allow gene expression. A popular model to explain looping suggests that CTCF functions with two subunits of a possible motor in a complex. The two subunits of the motor protein then slide along the genome in opposite directions, either actively (Sanborn et al., 2015) or through diffusion (Brackley et al., 2018), to grow or “extrude” this loop. The ring-shaped cohesin complex is a strong candidate for the motor as disruption of RAD21, a core component of complex, leads to loss of loop domains without affecting histone modification patterns. However, in the same study, a population of cohesin-independent loops was observed to be frequently anchored around enhancer enriched genomic regions (Rao et al., 2017). This suggests that in humans, in addition to CTCF and cohesin mediated loop extrusion, there is also an independent mechanism at play.

Topologically Associated Domains can further be part of long-range interactions resulting in chromosome compartments. The genome seems to be divided between A and B compartments. Based on epigenetic marks, the A compartment is enriched in actively transcribed chromatin and the B compartment in inactive chromatin. Dynamic changes in compartment organization and transcription support physiological state of the cell and organismal development (Lieberman-Aiden et al., 2009; reviewed by Eagen, 2018).

### Chromosome Territories and Radial Genome Organization

At the chromosome level, organization is specified in the form of preferential territories occupied by chromosomes within the nucleus (Figure 1). Typically, eukaryotic nuclei have heterochromatin concentrated at the periphery and around the nucleolus with gene rich chromosomes preferentially occupying a more central position (Cremer et al., 1993; Croft et al., 1999; Gilbert et al., 2004; Cremer and Cremer, 2006; Misteli, 2007), though there are also cell types with inverted chromatin organization (Solovei et al., 2009). Interestingly, mechanisms governing territorial chromosome organization seem to go a long way back in evolution. Preferential organization of gene rich chromosomes in the interior and gene poor chromosomes at the periphery is also observed in other primates (Tanabe et al., 2002) and radial organization of territories is observed all the way to the polyp *Hydra*, suggesting that specific radial positioning mechanism evolved at least 600 million years ago (Alexandrova et al., 2003). This radial genome organization pattern coincides with appearance of lamins that clearly parallel mammalian lamins in sequence conservation (Erber et al., 1999).

### Global Determination of Peripheral DNA for Radial Genome Organization

Studies showing the attachment of bacterial chromosome to the cell membrane (Jacob et al., 1963) and mitochondrial chromosome to the mitochondrial membrane (Yotsuyangi, 1966), suggested that membranes may not be merely a structural barrier to contain genomic material, but also a tethering point to stabilize/organize the genome. Accordingly, the nuclear envelope (NE) in eukaryotes is a major tethering point for specific genomic loci.

DamID facilitated the understanding of peripheral genome organization i.e., organization orchestrated by the NE. DamID maps, constructed by fusing bacterial Dam methyl transferase to a nuclear envelope protein, typically Lamin B1, enable the identification of global genome-NE interactions (van Steensel and Henikoff, 2000). Genome-NE interactions are interesting for two logical reasons – one, that such tethering theoretically stabilizes chromatin laying foundations for interphase chromosome topology and two, that tethered regions often show lower transcriptional activity.

Lamina Associated Domains (LADs), identified from DamID maps as stretches of the genome interacting with the periphery (Figure 1), are typically AT rich sequences, possess heterochromatin marks like H3K9me3 and H3K9me2 and overlap with the late replicating regions of DNA during S phase (Guelen et al., 2008). DamID over many different cancer cell lines and some cell types has revealed that certain LADs are invariant (constitutive) over the cell types sampled while others are cell type specific (facultative), with their release from NE often correlating with gene activation (Meuleman et al., 2013; van Steensel and Belmont, 2017). This suggests that generally, the nuclear periphery is a repressive environment, robustly anchoring over a third of the genome and so freeing up the nucleoplasmic volume required for crucial long-range regulatory interactions. The ability of LADs to allow or restrict certain TAD associations may be an important regulatory function of LADs beyond the sequestering of repressed genes at the NE (Robson et al., 2017). It is important to note, however, that, while gene repression from NE is the tendency in the cell lines most investigated, there are also many examples of gene activation at the NE (Van de Vosse et al., 2011). That the NE can be both repressive and activating suggests that at least part of how it functions in genome regulation is by creating an effective physical-spatial subcompartment in which either repressive or activating factors are sequestered/concentrated. If one considers the NE as a chromatin compartment by including the first 50 nm from the membrane, this volume would account for ∼1/40th of the total nuclear volume. It has already been shown that lamins and several nuclear envelope transmembrane proteins can bind both transcriptional repressors and transcriptional activators. Thus, depending on the affinities and NE proteins present in a particular cell type, the NE compartment would effectively increase the local concentration of these chromatin regulators to enhance either repression or activation (Heessen and Fornerod, 2007).

## *In silico* Modeling of Spatial Genome Organization

Several recent studies have attempted to approach the idea of genome organization from a more facile standpoint, using only the most basic components of the environment in which genetic material is contained to build models, rather than dealing with complexities that arose through evolution. Toward this end, a nucleus or a prokaryotic cell can be thought of as an environment with DNA (a polymer), DNA binding proteins and non-DNA binding proteins, all confined within a boundary.

Monte Carlo simulations yielding probability distributions for the range of possible outcomes to a problem are a useful computational tool to model genome organization. For simplicity, simulations were first performed to study only DNA-DNA interactions, considering two kinds of polymers in a confined space – flexible ones representing euchromatin and stiff ones representing heterochromatin. Remarkably, simulations showed that spontaneous movement of euchromatin to the interior and heterochromatin to the periphery had the greatest likelihood, recapitulating what is seen in cells. Additionally, stiff polymers exhibited greater contact frequencies, resulting in separation between rigid and flexible polymers (Cook and Marenduzzo, 2009). With the knowledge that heterochromatin is transcriptionally inactive and euchromatin is active, this separation resembles the idea that the genome is split into A (active) and B (inactive) compartments. It is important to note that within the confines of these simulations, merely entropic forces act on the genome.

Further, an additional layer of complexity was added to the system in the form of chromatin binding proteins, the system now involving DNA-DNA and DNA-protein interactions. This showed spontaneous aggregation of binding sites as a result of DNA-protein interactions. This promoted further binding of proteins from the soluble pool, in turn increasing local chromatin concentration. It is not hard to imagine then, that if the bound factors have bi- or multi-valency they can effectively bridge out forming chromatin loops that will remain stable for as long as the factors are bound. Such bound clusters and the resultant looping are stabilized again by a non-specific force called depletion attraction, which is in play in crowded environments like cells (Marenduzzo et al., 2006; Brackley et al., 2016).

In cells, there are many different transcription factors, each binding to DNA in a sequence specific manner. In such a situation, there would be specialized clusters, effectively separated in 3D space. Specificity can also be conferred by epigenetic marks separating euchromatin from heterochromatin. Thus, each individual transcriptional event would contribute its part in affecting global genome organization. This model of self-assembly and clustering is popularly referred to as the “transcriptional factor model” of genome organization (Hnisz et al., 2017; Cook and Marenduzzo, 2018).

*In silico* models collectively demonstrate how a certain degree of order is achieved in the system merely through entropic forces and stabilized by genome-wide transcriptional events.

## Changes in Genome Organization – a Developmental Timescale

Development is a complex process, involving extremely tight spatio-temporal regulation of gene expression. Dynamic physiological state-dependent changes in chromatin folding allow for this stringent regulation. With the understanding of hierarchical genome organization, we now explore its plasticity through organismal development.

### Gametes and the Totipotent Zygote

Multicellular life begins with fusion of haploid gametes to form a totipotent zygote which, through divisions, gives rise to a population of pluripotent cells which then differentiate into the many different lineages. 3D organization of the haploid genome within gametes was studied only recently. Hi-C data on mouse spermatozoa shows a remarkable conservation of TADs and loops, with those observed in somatic cells. There is a distinct enrichment of intrachromosomal contacts and contacts with DNA that is more than 10 Mb away (Battulin et al., 2015; Jung et al., 2017). However, such long range contacts can be merely due to the fact that DNA is packed within a 40 fold smaller volumetric space in a mouse sperm compared to an average liver cell (Ward and Coffey, 1991). While spermatozoa retain features of 3D genome organization of somatic cells, mouse oocytes show a dramatic loss of TADs and loops as they develop from transcriptionally active immature oocytes to transcriptionally inactive mature oocytes (Flyamer et al., 2017).

The genome of the zygote is transcriptionally inactive and utilizes gene products from the oocyte until a widespread recruitment of RNA polymerase II along with concurrent epigenetic changes leads to zygotic genome activation (ZGA) (Tadros and Lipshitz, 2009; Jukam et al., 2017; Hug and Vaquerizas, 2018; Xu and Xie, 2018). Microscopic evidence shows the shift from non-random to radial distribution of gene-rich regions after ZGA in bovine pre-implantation embryos (Koehler et al., 2009). In mouse zygotes, TADs are harder to detect but by the two- to four-cell stages TADs and compartments can be detected suggesting that re-establishment of genome organization is concurrent with ZGA (Gassler et al., 2017; Ke et al., 2017). ZGA is triggered by transcription factors such as Zelda in Drosophila (Liang et al., 2008), Nanog, Pou5f1 and SoxB1 in zebrafish (Lee et al., 2013; Leichsenring et al., 2013) and Oct4 (homolog of Pou5f1) in humans (Gao et al., 2018). These pioneer transcription factors compete with histones to bind to and maintain open chromatin states to facilitate recruitment of other factors during ZGA (Veil et al., 2019) and this establishes the chromatin state of pluripotent cells.

### Pluripotency

Pluripotent cells can self-renew indefinitely and have the potential to differentiate into nearly any lineage of a mature organism. These cells are interesting for studies on early developmental events and for their therapeutic potential. Most of our current understanding of pluripotency comes from *in vitro* cultures of embryonic stem cells (ESCs) derived from the inner cell mass (ICM) of the blastocyst. These cells are maintained in their pluripotent state through the function of core transcription factors like Oct3/4, Sox2, and Nanog, which orchestrate self-renewal by repressing expression of genes required for lineage commitment and/or sustaining expression of one another (Williams et al., 1988; Chambers and Smith, 2004; Niwa, 2007; Ema et al., 2008). Whether pluripotent cells themselves have a special genome architecture has been of interest for many years. Globally, pluripotent cells have loosely packed hyperdynamic chromatin with poorly defined heterochromatin (Ahmed et al., 2010; Mattout and Meshorer, 2010). Widespread transcription of coding regions and repetitive elements shows the openness of chromatin in ESCs (Efroni et al., 2008). Higher order chromatin architecture of pluripotent cells is uniquely shaped by transcription factors like Oct4 and Nanog. Genomic clusters of pluripotency factor binding sites find other distally located clusters rather easily, increasing the local density of binding sites. This spatial clustering is governed by transcription factors themselves and leads to efficient transcription of nearby genes (de Wit et al., 2013).

The NE being a major tethering platform and generally serving as a transcriptionally repressive environment contributes to tissue-specific genome organization but whether it has a role in maintaining genome organization of pluripotent cells is an interesting concept to explore. In most differentiated mammalian cells, the lamina, a meshwork formed under the NE, composed of typically 2–6 splice products of the three lamin genes (A, B1 and B2), plays a major role in determining genome interactions with NE, along with several other nuclear envelope transmembrane proteins (NETs). Immunofluorescence studies show the absence of Lamin A/C until tissue specification begins at embryonic day 12 during mouse embryogenesis (Röber et al., 1989) and accordingly, Lamin A/C has been used as a marker for *in vitro* differentiation in many studies (Constantinescu et al., 2006). The proclaimed absence of Lamin A/C has also been used to explain chromatin dynamics and nuclear plasticity of ESCs (Melcer et al., 2012). However, more recent studies suggest that reports of its absence were likely mistaken due to epitope masking. In mouse ESCs (mESCs), Lamin A/C expression was shown to be present using three different antibodies in immunoblots over 9 independent ESC lines. Lamin A/C expression and localization to the NE is also seen in the ICM cells of the blastocyst (Eckersley-Maslin et al., 2013). Furthermore, it has also been shown that Lamin A/C localization at the NE depends on Lamin B1 in mESCs (Guo et al., 2014). These reports conclusively show that Lamin A/C is expressed and recruited at the NE in mESCs and absence of this protein is not an appropriate marker for pluripotency.

Mouse ESCs depleted of A- and B-type lamins, and subjected to Emerin-DamID, yielded LAD profiles similar to LaminB1-DamID, suggesting that lamins are not necessary in establishing genome-NE interactions during pluripotency (Amendola and van Steensel, 2015). One caveat in this study is that Lamin A/C depletion achieved through RNA interference only guarantees substantial decrease, not complete abolishment. Another mESC study with deletions in all three lamin genes yielded a decondensation of constitutive heterochromatin and detachment of facultative heterochromatin. There was no increase in overall transcription but the detachment of chromatin affected 3D genome organization enough to affect inter-TAD interactions without compromising overall TAD structures. This suggests a role for lamins in maintaining 3D genome organization in mouse ESCs (Zheng et al., 2018).

### Differentiation

Differentiation of pluripotent cells is coupled with a dramatic reorganization of the genome. Typically, movement of a genomic locus toward the periphery or its direct tethering to the NE leads to its repression while movement toward the nuclear interior often results in its activation. At the chromosome territory level, ESCs have no special organization compared to differentiated cells. However, a comparison between pluripotent human ESCs (hESCs) and differentiated lymphoblastoid cells revealed a more internal position for the locus encoding NANOG in the nuclei of ESCs where it is expressed highly. Similarly, OCT4 shifted in localization from the interior of its chromosome territory in hESCs to the surface of the territory in lymphoblastoid cells (Wiblin, 2005). Such observations were corroborated in a recent study using three chromosomes that differ in size and gene density and also contain loci encoding pluripotency and lineage regulators like NANOG, OCT4, and CDX2. Radial position of these chromosomes remains unchanged during bovine development from a zygote into a blastocyst. However, the loci for both, NANOG and CDX2, were shown to relocate to the surface or outside the chromosome territory when stage specific expression was required. This relocation was accompanied by a change from regular to irregular territory shape (Orsztynowicz et al., 2017). Additionally, HoxB locus, containing genes that control body plan of the embryo during development, decondenses during retinoic acid induced differentiation of mESCs. This decondensation is concurrent with the movement of HOXB1 and HOXB9 from the interior of their chromosome territory to its periphery in synchrony with their expression profiles (Chambeyron and Bickmore, 2004).

**FIGURE 2:**
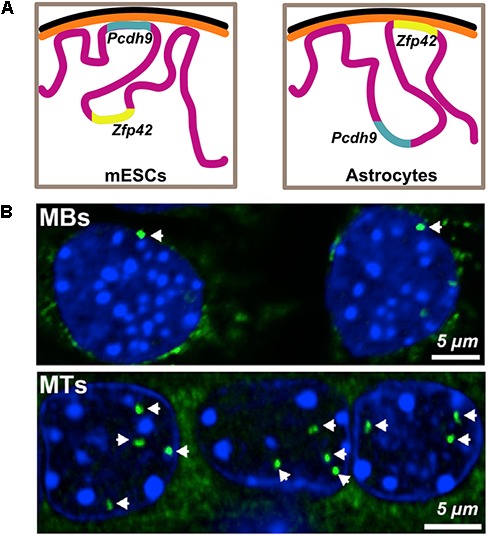
Functional basis for relocation of genes during differentiation. **(A)** Pluripotency factor Zfp42 is found in the nuclear interior in mouse embryonic stem cells (mESCs) and is later sequestered to the periphery upon neuronal differentiation. Pcdh9, an important component of neuronal junctions in the brain, is tethered and therefore repressed during pluripotency and is later released, concurrent with its activation in astrocytes. Illustration is based on data from Peric-Hupkes et al., 2010. **(B)** Fluorescence *in situ* hybridization images showing the relocation of Ttn locus (green) encoding Titin, an important protein for muscle differentiation, from the nuclear periphery to the interior upon differentiation of myoblasts (MBs) to myotubes (MTs). Data reproduced from Robson et al., 2016 according to the Creative Commons Attribution License (CC BY) (https://creativecommons.org/licenses/by/4.0/).

A more detailed study looking at overall changes in peripheral genome organization using DamID in pluripotent mESCs, neural progenitors (NPCs) and terminally differentiated astrocytes shows the relocation of several developmentally important genes. The release of neuron specific *Pcdh9* is concomitant with its transcriptional activation in the neural lineages and the tethering of *Zfp42*, a pluripotency marker, with its repression as lineage specification progresses (Peric-Hupkes et al., 2010; Figure 2). Within the nucleus, higher-order chromatin structures also change during differentiation. A comparison of Hi-C data from hESCs with four other human-ES-derived lineages shows that ∼36% of the genome switches between A and B compartments during differentiation. Notably, there appears to be an expansion in the B compartment that typically contains inactive heterochromatin. Interestingly, regions that switch compartments typically correspond to a single TAD, suggesting TADs reposition as entire units. Consistent with studies showing repositioning of individual genes, a switch from A to B compartment was accompanied with reduced expression of the genes (Dixon et al., 2015). Analogously, during *in vitro* differentiation of mESCs into NPCs and eventually cortical neurons, there is a decrease in long-range A-compartment interactions and a concomitant increase in inactive B-type domain interactions, suggesting formation of heterochromatin. Finally, cell-type specific enhancer-promoter interactions were also detected, supporting the model that dynamic chromatin looping from enhancers results in gene activation (Bonev et al., 2017). These observations were corroborated in a recent study looking at higher order chromatin during *in vitro* adipogenesis and myogenesis, where they also show that marker genes maintain an active chromatin state by either remaining in the A compartment or switching from B to A compartment during differentiation (He et al., 2018). These studies collectively illustrate that genome organization is dynamic during development, with a functional basis for repositioning of certain important loci.

Tissue-specific gene expression is in part achieved through a differential composition of the NE itself. Although the main structural components of NE seem to be fairly constant across cell types, there is a dynamic remodeling in its composition in response to various physiological processes ranging from cell division to differentiation (reviewed in detail by Gruenbaum and Foisner, 2015; Ungricht and Kutay, 2017). The INM in particular, shows enormous tissue diversity in its composition with the presence of hundreds of nuclear envelope transmembrane proteins (NETs). Several of these NETs bind to lamins and to chromatin proteins (Wong et al., 2014). A comparison of the NE proteome from a lymphocyte-enriched fraction of blood leukocytes, muscle and liver showed that less than 20% of the NETs were shared between all three tissue types, highlighting their tissue-specific expression patterns. Interestingly, the higher the degree of tissue-specificity in NETs, the more recent their appearance in evolution, with variation observed even within closely related organisms, suggesting that tissue-specific NETs evolved to enable functional diversity of tissues (Korfali et al., 2010; Wilkie et al., 2011; Korfali et al., 2012; de las Heras et al., 2013). Several of these NETs have been tested for and have shown to be able to affect repositioning of entire chromosomes (Zuleger et al., 2013). Muscle-specific NETs TMEM38A, WFS1, and NET39 directly affected repositioning of myogenic genes like *Nid1*, *Ptn*, *Cxcl1*, etc. Additionally, these NETs were also important for myotube formation (Robson et al., 2016). Analogously, adipocyte specific NETs TMEM120A and B are essential for proper adipogenesis (Batrakou et al., 2015). Several nucleoporins (Nups) also show tissue-specific expression patterns, along with an ability to drive differentiation. In addition to their canonical function in nucleocytoplasmic transport, Nups have also been shown to directly bind to specific genomic locations, with an ability to affect gene expression by promoting stronger transcription or by altering chromatin structure (Sood and Brickner, 2014; Talamas and Capelson, 2015).

### Senescence

Cellular senescence, a state of irreversible growth arrest, is a hallmark of organismal aging. Senescence is accompanied by a striking reorganization of the genome, detected by the presence of DAPI dense senescence associated heterochromatic foci (SAHF) seen in the nuclear interior with the corresponding loss of LADs from the periphery. SAHF are ordered structures with a core enriched in constitutive heterochromatin, surrounded by facultative heterochromatin and the outermost layer being euchromatin, a complete reversal of organization seen in healthy replicating cells. SAHF are devoid of active transcription sites with many cell-cycle genes sequestered within them to prevent these cells from cycling (Mehta et al., 2007; Parry and Narita, 2016). The characteristics of senescence appear to be much the same in different cell types and formation of SAHF is suggested to be the end-point in chromatin remodeling during differentiation, with somatic cells being the intermediate state (Chandra et al., 2015).

## Mis-Regulation of the Genome Causes Diseases

Disease-linked single nucleotide polymorphisms (SNPs) in protein coding regions can alter protein structure and function, thus it is easy to explain how they can cause diseases. However, over 90% disease-associated SNPs are found outside protein coding transcripts, highlighting the importance of non-coding RNAs and other regulatory elements such as promoters and enhancers in maintaining homeostasis (Khurana et al., 2016; Lekka and Hall, 2018).

Mutations like deletions, inversions, duplications and translocations cause structural variations (SVs) in the genome thereby disrupting higher order chromatin structures like TADs along with effects on gene expression and dosage (Spielmann et al., 2018). Oncogenic cells often accumulate SVs through their progression and retain the ones that confer fitness advantage through their somatic evolution. In prostate cancer cells, additional TAD boundaries are formed at the site of *TP53* deletion, leading to an overall decrease in TAD sizes, affecting 3D organization at a global level (Taberlay et al., 2016).

Germline SVs with microdeletions and microduplications are implicated in congenital diseases (Cooper et al., 2011; Soemedi et al., 2012). Accurate gene expression patterns and polarities during embryonic development are achieved through tight regulation by cis-regulatory elements called enhancers, which activate transcription through physical contacts with gene promoters over long distances. Enhancer activity is both tissue specific and temporally controlled (Spitz and Furlong, 2012). SVs causing disruption in either their expression or activity cause congenital diseases. Limb development is a well-studied developmental program and presents examples elucidating the importance of enhancer activity. Sonic Hedgehog (encoded by the gene *Shh*), an important morphogen specifying the number of digits and digit identity during limb morphogenesis is controlled by *Zrs*, its enhancer. Gain-of-function mutations in *Zrs* lead to SHH expression at ectopic sites causing preaxial polydactyly, whereas an inversion leading to its loss-of-function causes holoprosencephaly along with upper limb syndactyly and lower limb polydactyly (Lettice et al., 2011).

**FIGURE 3:**
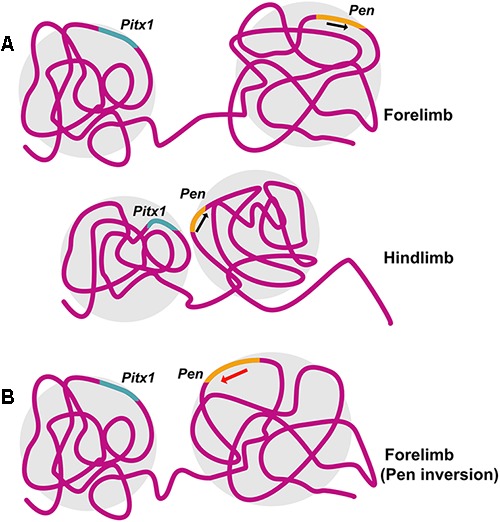
Structural variation can lead to disruption in chromatin conformation and disease. *Pitx1* specifies hindlimb identity in mouse. **(A)** 3D chromatin conformation in mouse forelimb prevents interaction of *Pitx1* with its enhancer *Pen*, leading to preferential expression of *Pitx1* in the hindlimb. **(B)** Introducing an inversion in the locus containing *Pen* results in a structural variation that leads to a hindlimb-like 3D chromatin conformation in the forelimb allowing *Pitx1-Pen* interaction and mimicking arm-to-leg transformation observed in Liebenberg syndrome. Illustration is based on the study conducted by Kragesteen et al., 2018.

Structural variations including inversion, duplication and deletion in the TAD containing *Epha4*, a gene important normal innervation of the limb, lead to ectopic promoter interactions and cause different kind of syn- and polydactyly (Lupiáñez et al., 2015). *Pitx1*, a gene important for hindlimb identity, is regulated by its enhancer *Pen* which is active in both forelimb and hindlimb. However, it is the difference in 3D chromatin conformation between forelimb and hindlimb that preferentially allows *Pen-Pitx1* interaction in the hindlimb, while constraining it in forelimb. A perturbation of the 3D genome, achieved by inversion of the enhancer element led to conversion of the inactive 3D conformation of the forelimb into an hindlimb-like active conformation, PITX1 expression and an arm-to-leg transformation with an ectopic patella formation in the arm, as seen in Liebenberg syndrome in humans (Kragesteen et al., 2018; Figure 3).

Lamins and several NETs with functions in genome organization are also linked to a wide range of diseases generally termed laminopathies or nuclear envelopathies. Lamin mutations have been implicated in premature aging phenotypes like The Hutchinson Gilford Progeria Syndrome as well as muscular dystrophies, lipodystrophies, dermopathies, neuropathies and cardiomyopathy (Rankin and Ellard, 2006). The effects of muscle-specific NETs on both the repositioning of critical genes during myogenesis and their knockdown inhibiting myogenesis led to the suggestion that the tissue-specific NETs might mediate the tissue-specific pathologies of some of these diseases caused by mutations in the more widely expressed NETs (Robson et al., 2016). In keeping with this idea, it is interesting that mutations in some tissue specific NETs that have roles in genome organization have been linked to tissue-specific pathologies. For example, mutations in the gene encoding WFS1, a NET preferentially expressed in muscles and the retina, causes Wolfram Syndrome characterized by optic atrophy among other phenotypes (for detailed review on how nuclear membrane diversity can contribute to diseases, please see Worman and Schirmer, 2015). In all these cases, alteration of the 3D genome in response to disruption of nuclear architecture leads to mis-regulation of gene expression and disease.

## Conclusion

Complexity of the genome increased concurrently with evolution toward multicellularity. Along with this complexity the number of potential molecular players involved in orchestrating genome organization also increased. While the “loop extrusion model” elaborating the importance of CTCF and cohesin in maintaining architecture and “transcription factor model” emphasizing spontaneous formation of specialized clusters due to transcriptional events seem like two schools of thought, we believe that the two are not exclusive.

A first-degree organization can be achieved merely by specific DNA-protein and protein-protein affinities and driven by entropy, as shown by *in silico* modeling data. Transcription is a common denominator in all kinds of organisms containing DNA as their genetic material. That transcription itself should drive overall chromatin architecture is almost first principle and a simplistic explanation for the seemingly elaborate problem that is genome organization. However, architectural proteins like CTCF and cohesin confer stability to this order and might facilitate genome reorganization, aiding in accurate spatio-temporal gene-expression during development. In fact, deletion of CTCF binding sites has been shown to alter cell fate decisions (Narendra et al., 2016). It was recently also shown that during transcription elongation, RNA polymerase II displaces cohesin from CTCF sites leading to local decompaction. Conversely, inhibition of elongation leads to cohesin accumulation at previously transcribed CTCF sites thereby mediating chromatin looping and 3D genome architecture (Heinz et al., 2018). This study elegantly demonstrates how the two mechanisms are neither exclusive nor incompatible.

Topologically Associated Domain boundaries are defined by CTCF. Loop extrusion requires cohesin loading onto CTCF sites to facilitate its motor function. The distribution of cohesin in the mouse genome is governed by WAPL, a cohesin unloading factor. Interestingly, WAPL also controls the length of loops extruded by CTCF, suggesting that the longer cohesin is bound to the DNA, the longer the loops are (Busslinger et al., 2017; Haarhuis et al., 2017). While CTCF has a crucial and instructive function in chromatin folding, its depletion by auxin-mediated degradation does not cause large-scale gene expression changes up to 24 h, demonstrating a weaker role for CTCF in maintaining or facilitating gene expression. A similar depletion of nearly all DNA-bound cohesin complexes also revealed an acute loss in contact domains stabilized by CTCF and cohesin but minor changes in gene expression profiles (for detailed review see Rada-Iglesias et al., 2018). Furthermore, the active and inactive compartments of the genome also remain properly segregated post CTCF depletion, suggesting that compartmentalization is independent of TAD formation (Nora et al., 2017). All these studies strongly suggest that our understanding of the molecular mechanisms governing genome organization is still limited.

**FIGURE 4:**
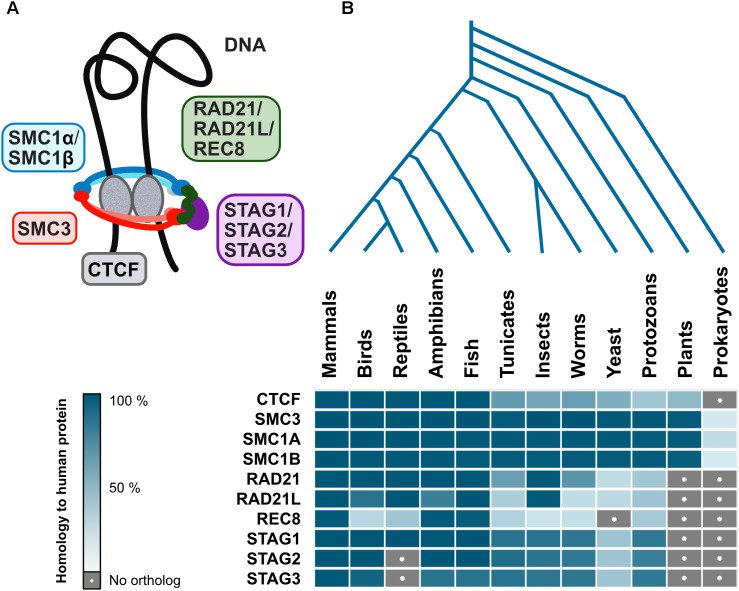
Evolutionary conservation of architectural proteins. **(A)** Vertebrate cohesin complex is a multi-subunit protein complex made up of a dimer of SMC proteins (SMC3-SMC1α/β) which is the core structural component forming a closed ring along with a kleisin (RAD21/RAD21L/REC8) and STAG1/STAG2/STAG3. The cohesin complex acts in conjunction with CTCF, an architectural protein found at TAD boundaries, to facilitate DNA looping **(B)** Human genome architectural proteins were queried against NCBI’s non-redundant protein sequence database, using BLASTP with default parameters, for 140 organisms covering all major taxonomic divisions. A representative for each was then selected: *Mus musculus* (mammals), *Gallus gallus* (birds), *Anolis carolinensis* (reptiles), *Xenopus laevis* (amphibians), *Danio rerio* (fish), *Ciona intestinalis* (tunicates), *Drosophila melanogaster* (insects), *Caenorhabditis elegans* (worms), *Saccharomyces cerevisiae* (yeast), *Paramecium tetraurelia* (protozoans), *Arabidopsis thaliana* (plants), and *Escherichia coli* (prokaryotes). When matches were obtained, the percent of the query (human protein) that has significant homology to the target protein were displayed as a heatmap in blue shades (dark = high homology, pale = low homology). Gray with a white dot indicates no matches were found.

Interestingly, cohesin complex is evolutionarily conserved among eukaryotes, with varying degrees of similarity for different subunits, however, CTCF is not as well conserved (Figure 4). Although there are proteins with a certain degree of homology to human CTCF in plants and lower eukaryotes, this is mostly due to similarities to other zinc finger proteins which may have wildly different functions. However, *Drosophila* have CTCF and also other insulator proteins, suggesting that at least some invertebrate branches will have CTCF too (Van Bortle et al., 2012). While a CTCF homolog has not yet been described in yeast or plants, ectopically expressed vertebrate CTCF is functional as an insulator in *S. cerevisiae* (Defossez and Gilson, 2002), indicating that the mechanism is already in place, albeit governed by other yet-to-be-described proteins. Even prokaryotic chromosome is organized into TADs resembling those in higher organisms (Le et al., 2013), further suggesting that the mechanism for such organization indeed goes back a long way in evolution. Finally, ubiquitous presence in eukaryotes and prokaryotes of SMC (structural maintenance of chromosomes) proteins that form the core of the cohesin complex (Figure 4; Hirano, 2006) reaffirms the idea that architectural proteins stabilizing genome organization is also as basic a mechanism as formation of specialized clusters in response to transcriptional events.

We propose that genome organization is perhaps as old as DNA itself, firstly forming specialized clusters driven by entropic forces, and secondly stabilizing those structures with architectural proteins that facilitate higher order interactions. While the basic mechanism has remained conserved through evolution, individual proteins involved may have diversified and only recently we have started identifying the players.

Evolution, however, is an on-going process and the idea of different mechanisms and molecular players orchestrating and maintaining genome organization is not eccentric. Dinoflagellates have a radically different genome architecture from other eukaryotes in that they have permanently condensed chromosomes that lack nucleosomes (Gornik et al., 2012). In mammals alone, nuclei of rod cells of the nocturnal mammalian retinas show an inverted pattern of organization with heterochromatin concentrated in the interior and euchromatin at the periphery (Solovei et al., 2009). Simulations show that this inverted organization helps these nuclei act as collecting lenses to efficiently channel light, suggesting that genome organization could also achieve purposes other than regulation of gene expression (Błaszczak et al., 2014). Thus, different mechanisms may yield different architectures, depending on which form facilitates function most efficiently offering a fitness advantage during evolution.

## Author Contributions

AS and JIH wrote the manuscript and made figures with help from ECS.

## Conflict of Interest Statement

The authors declare that the research was conducted in the absence of any commercial or financial relationships that could be construed as a potential conflict of interest.
